# Deliver Us from Our Friends

**Published:** 1893-07

**Authors:** 


DELIVER US FROM OUR FRIENDS.—Lightning Love, of the Magnificent *Medical Mirror*, defends his friend, the quandam editor of the *Weekly Medical Review*, in the following equivocal manner. Reproducing our article, "The Free-booter Again," in which we charged the *Weekly Medical Review* with repeated piracy on our columns, and its editor, Ohman Dumesnil (provokingly set up by our compositor and appearing in first "proof" as "old man Damsneak"), with playing us a shabby trick, the *Mirror* indulges in the following "reflections:"

"We would remark, for the information of Brother Daniel, that Dumesnil is a good fellow. He does not mean to play shabby tricks, but then you know, when a man hires himself to a journal such as the one referred to, which is nothing more than the official organ of a proprietary medicine company, even though he be as good a man as Dumesnil, particularly with a proprietor that puts into his journal what he pleases, in spite of the editor, he may sometimes be made to appear worse than he is."

This reminds us of the old fellow whose son was convicted of larceny. He said, "Jimmy's a good boy, but he *will steal.*" Love would throw the blame on the publishers of the *Review*, forgetting the editor's letter in which he *claimed the right to steal*, alleging that it was a "custom."

